# Transforming the automotive industry: Defining, clustering, and efficiency analysis of the next-generation automotive ecosystem

**DOI:** 10.1371/journal.pone.0343135

**Published:** 2026-02-23

**Authors:** Minjung Shon, Joon Kim, Hongbum Kim

**Affiliations:** 1 Department of Economics, Major in Intellectual Property, Kyonggi University, Suwon-si, Gyeonggi-do, South Korea; 2 Korea National Industrial Convergence Center, Korea Institute of Industrial Technology, Ansan-si, Gyeonggi-do, South Korea; 3 College of Business, Gachon University, Seongnam-si, Gyeonggi-do, South Korea; Industrial University of Ho Chi Minh City, VIET NAM

## Abstract

Using firm-level data, this study explores how next-generation automotive innovations are reshaping the automotive industry. The sector is now transitioning from a previously defined vertically integrated supply chain into a more horizontal ecosystem in which multiple companies collaborate to build next-generation vehicles. To define and categorize this ecosystem, this study classifies firms into three major groups of electric vehicles (EVs), autonomous vehicles (AVs), and general automotive technologies (GATs) by employing K-means clustering based on patent data. This study then measures firm efficiency in each ecosystem using meta-frontier analysis to compare their technical efficiency over the periods 2017–2019 and 2020–2022. The results show that the EV ecosystem led the initial growth while the GAT ecosystem continued to make steady progress. Later, the AV ecosystem exhibited remarkable technological innovation and efficiency gains. These findings indicate that efficiency dynamics and technology gaps differ systematically across ecosystems, reflecting heterogeneous innovation trajectories within the broader industrial transformation. Overall, our findings clarify the growth potential and trajectories in the automotive industry, thereby providing insights for stakeholders seeking to navigate this rapidly changing landscape and contributing to a clearer understanding of the next-generation automotive ecosystem.

## 1. Introduction

Automotive production centers on original equipment manufacturers (OEMs), which collaborate with vehicle frame manufacturers and component suppliers. However, an internal combustion engine (ICE) vehicle consists of 25,000–30,000 individual parts, and thus, handling the entire production process alone is difficult for the final manufacturer to handle. Consequently, close inter-firm collaboration is essential. Thus, the automotive industry is comprised of a robust supply chain (SC) that consists of OEMs responsible for design, production, and distribution; multi-tier component suppliers; and related service businesses [1–3]. Specifically, the component SC is organized into several tiers. Tier 1 suppliers interact directly with the OEM to provide key components, while tiers 2 and 3 produce sub-components and supply them to tier 1 in turn. This arrangement not only allows for modular production and utilization, but also reduces capital demand and provides flexibility in business operations to respond to market and technological changes.

During the last several decades, the automotive industry has shifted from the aforementioned traditional vertical hierarchical structure to a more horizontal industrial ecosystem. This shift has been driven by the rising collaboration among diverse stakeholders and restructuring of value networks to enhance innovation outcomes [4–6]. Having dominated the industry for the past century, ICE vehicles are increasingly becoming digitalized and environmentally oriented owing to advancements in information and communications technology (ICT) and convergence technologies [6–10]. Electric and hydrogen fuel cell vehicles exemplify this transition toward eco-friendly, while digitalization is evident in autonomous and connected cars. These new vehicle types are restructuring the traditional ICE vehicle-based SC to focus on ICT, software, and green technologies. The simplification of component SCs and strengthening of internal technological capabilities are subsequently promoting role restructuring within the value network (e.g., Tier 1 suppliers providing integrated solutions) [11–13].

With the development of this next-generation automotive industry, the scope of the relevant companies has expanded. The boundaries between producers and consumers, small and medium-sized enterprises and large corporations, online and offline markets, and products and services are disappearing in the era of industrial convergence. Firms with competitive technologies can operate actively across multiple domains. For example, current and emerging automobiles are evaluated by both their ability to facilitate communication between people and cars, cars themselves, and people, as well as their mechanical performance. Most vehicle functions can now be accessed via electronic devices such as computers and tablets, which makes in-vehicle infotainment an important attribute. Consequently, ICT and software firms are actively penetrating the automotive sector [14–16].

Despite the fact that numerous companies are developing technologies and engaging in convergence activities to drive consumers’ adoption of these new vehicle types, the growth of the relevant markets can be stagnant or delayed owing to technological, social, regulatory, and market obstacles. For example, autonomous vehicles (AVs) must contend with safety, ethics, and liability, while electric vehicles (EVs) face challenges such as price competitiveness, charging infrastructure, driving range, and safety issues. In this context, how the competitiveness of the automotive industry is changing in response to the paradigm shift warrants analysis. The industry has made substantial investment to convert ICE vehicles to digitalized and eco-friendly devices and the related market is being established; thus, how ecosystems are being shaped is a worthwhile avenue of investigation.

Accordingly, the following research questions can be raised. First, how can the next-generation automotive ecosystem be objectively defined and categorized based on firm-level technological activities? Second, how different is the growth potential and development trajectory for each ecosystem over time? To address these questions, this study first categorizes firms in automotive SC into the next-generation automotive ecosystems by an unsupervised learning approach based on patent data. Next, this study measures firm efficiency in each ecosystem using stochastic frontier and meta-frontier analyses, and then conducts a comparative analysis over different periods to examine overall industry competitiveness.

The significance of this study is as follows. First, previous studies have mainly investigated efficiency in only one vehicle segment using data envelopment analysis (DEA) or stochastic frontier analysis (SFA) [17–19]; the current study applies a meta-frontier analysis (MFA) approach to enable cross-sectional comparisons across different vehicle groups over the same period. Firms’ efficiency can be examined via traditional approaches including DEA and pooled SFA. However, when the production function of one group of firms clearly differs from that of another, direct comparison of their efficiency becomes challenging. To overcome this limitation, we adopt MFA, which uses a meta-frontier production function that envelops different production functions and enables comparisons among them [20–22]. MFA has been used to compare the effects of different technologies, strategic decisions, and policy treatment groups [23–28], particularly the different directions of industrial ecosystems and comparing their growth. Second, the current study uses patent data and conducts K-means clustering to categorize groups exhibiting sufficiently heterogeneous production functions to clarify the existence of different production functions across these ecosystems. Rather than conventional standard industrial classification codes or subjective categorization, the use of technologies possessed by firms in the clustering technique enables clear and objective categorization within the next-generation ecosystem, where boundaries are becoming increasingly blurred due to industrial convergence. Third, we adopt firm-level data obtained from the automotive ecosystem in South Korea, which has emerged as a major contributor to the nation’s industrial growth over the past several decades [29,30]. Hence, South Korea’s automotive industry can serve as a notable case for our analysis [31,32]. Furthermore, the country’s rapid transition to next-generation vehicles through continuous technological innovation can offer valuable implications for nations seeking to enhance their own automotive sectors.

The remainder of this paper is organized as follows. Section 2 reviews the relevant literature and presents the research context. Section 3 describes the methodology and models used to estimate the efficiency of the next-generation automotive industry in South Korea. Section 4 discusses the estimation results. Finally, implications and conclusions are provided in Section 5.

## 2. Literature review

### 2.1. Definition of next-generation vehicles

In all industries, digital transformation and technological innovation are being driven by advances in big data and artificial intelligence (AI). This includes the automotive industry, which is transitioning to next-generation vehicles such as EVs, AVs, connected cars, and hydrogen-powered vehicles, consequently leading to increasingly specialized and diversified segments. The CASE framework (connectivity, autonomy, sharing, and electrification) typically summarizes these developments [33–35].

Connectivity refers to vehicles’ ability to interact with each other via the Internet, the surrounding environment, and various digital networks to significantly enhance vehicle functionality and user experience [36–38]. Advances in vehicle connectivity have led to autonomous driving, as technologies that connect systems and digital devices are essential for developing fully AVs [39–41]. As the Internet of Things (IoT) has become more prevalent in our daily lives, connected car technology has also evolved [42]. Previous studies have included conceptual discussions on definitions, problems, solutions, and trends regarding connected cars [37,42,43]. Studies have focused on security aspects and vulnerabilities that are important to such vehicles [44–46] and investigated data governance and technology demonstration based on the collected data [43,47,48].

AVs can sense their surroundings, comply with traffic regulations, and identify optimal driving routes to operate without human control [49,50]. Firms pursue fully autonomous driving as their ultimate goal through the integration of cutting-edge technologies, including IoT-based sensors, mobile- and network-based communications, big data, and AI. Ongoing research covers a wide range of subtopics: sensor and data collection, autonomous driving algorithm development, communication technologies, human–machine interaction, safety and its validation, ethical and legal issues, social impacts, and economic analysis [51–55].

Car-sharing has attracted scholarly attention from economic, environmental, technological, and social perspectives via an integration of the sharing economy principles into vehicles. From a technical perspective, vehicle allocation and operation optimization, scheduling, and dispatching algorithms have received attention [56,57]. From a social and economic perspective, research has focused on user behavior, demand forecasting, diffusion, fairness and accessibility, privacy, and data security issues [26,58–60]. Additional research has explored topics such as how car-sharing can reduce carbon emissions and congestion, whether it can create synergies when combined with other next-generation vehicle technologies, and can guide regulatory and policy measures [61]. Implementing car-sharing can enable societies to address various social issues while maximizing the economic, environmental, and technological benefits [62].

Owing to the government’s commitment to reducing carbon emissions, lowering maintenance costs, and enhancing price competitiveness, as well as the increasing consumer adoption of such vehicles and rising number of environmental regulations, the market share of EVs is growing [63–65]. Research on EVs can be broadly categorized into energy management and optimization [66–69] and technological developments (power electronics, battery systems, and charging infrastructure). These technology-focused studies provide insights into EV components and subsystems that can help clarify the industry ecosystem.

Overall, the next-generation automotive industry is distinguishable from the traditional one centered on ICE vehicles. However, given that innovation continues to reshape this sector, creating clear boundaries in terms of components, technologies, and operations is challenging. The current study focuses on two major next-generation automotive domains, EVs and AVs, to identify the subsectors within these domains and conduct further analysis on the structure and efficiency of the ecosystem.

### 2.2. Changes in the next-generation automotive industry

**Fig 1:**
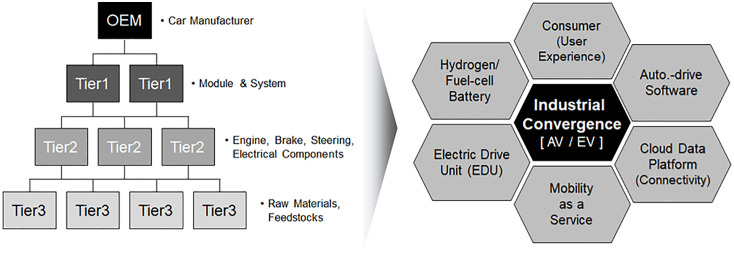
The shift from vertical to horizontal ecosystems. *Note*: OEM: original equipment manufacturer; AV: autonomous vehicle; EV: electric vehicle.

As illustrated in Fig 1, the shift from a vertical SC to a horizontal ecosystem in the automotive industry is fundamentally rooted in innovation system theory, which emphasizes the use of collaborative networks among firms, academia, and governments to drive technological advancement [70,71]. Thriving on open innovation, knowledge sharing and collective problem-solving in this horizontal ecosystem are accelerating the diffusion of CASE technologies (e.g., joint R&D in battery technology and autonomous driving algorithms). By aligning stakeholder incentives and reducing fragmentation, institutional frameworks, including government policies (e.g., EV subsidies and AV safety regulations) and standardization initiatives (e.g., vehicle-to-everything (V2X) communication protocols), are further shaping the ecosystem’s evolution [4,72]. For instance, Tesla’s open-source patent strategy exemplifies how innovation systems foster cross-industry collaboration, enabling smaller firms to contribute to electrification while strengthening the competitiveness of the overall ecosystem [73].

From a value network theory perspective, the horizontal ecosystem reflects a reconfiguration of value creation through modularization and specialization [74,75]. Tier 1 suppliers, which were once confined to manufacturing discrete components, now provide integrated solutions (e.g., Bosch’s autonomous driving systems), ultimately blurring the traditional boundaries between OEMs and technology providers. Simultaneously, data-driven services such as predictive maintenance enabled by connected car telematics create new revenue streams, thereby transforming vehicles into platforms for continuous service delivery [9,76]. This restructuring reduces reliance on established SCs, instead prioritizing flexible partnerships that align with dynamic market demand. For example, Hyundai’s collaboration with Aptiv on autonomous driving software emphasizes how value networks prioritize expertise over vertical integration, thus enabling firms to focus on their core competencies while leveraging external innovations.

As well as a technological trend, the CASE framework is also a catalyst for systematic change. The connectivity of vehicles (e.g., 5G-V2X systems) relies on interoperable standards developed through cross-sector alliances such as the Automotive Edge Computing Consortium [36,37]. Similarly, autonomy demands collaboration among sensor manufacturers, AI developers, and regulatory bodies to address ethical and safety challenges [51]. Furthermore, the sharing economy model disrupts ownership paradigms, consequently necessitating partnerships between OEMs and mobility platforms (e.g., GM’s Maven) to optimize fleet utilization [56,62]. To ensure sustainable scaling, electrification hinges on ecosystem-wide coordination, from raw material suppliers (e.g., lithium miners) to recycling firms [63]. The theoretical lens of embedding innovation systems and value networks into the CASE framework can help analyze how these changes are redefining competitiveness in the automotive sector.

### 2.3. Efficiency analysis in the automotive industry

Studies on the automotive industry’s efficiency differ in their methodological approaches and their categorization of the subgroups in the sector. The literature can be divided based on two main methodological approaches: SFA and DEA. The former assumes a specific functional form and adopts econometric methods to estimate unknown parameters from input and output data. This approach explicitly models inefficiency and random error terms, which allows for statistical significance tests to be conducted. Conversely, the latter approach constructs a non-parametric frontier from observed data and then compares this with actual data to measure efficiency. DEA does not require a specific functional form and can handle multiple input and output factors; however, as this method cannot distinguish between technical inefficiencies and statistical errors, DEA cannot provide significance tests for its estimates.

Neves et al. [77] used DEA to compare the efficiency of 20 European countries in expanding their battery EV markets and determined that renewable energy development affected national efficiency. To classify vehicles as efficient or inefficient, Prakash and Mohanty [78] collected 50 decision-making units of eco-friendly car manufacturing and suggested several approaches to improve efficiency. Additional studies on efficiency have further demonstrated that efficiency levels vary by a country’s vehicle configurations [79–81]. Furthermore, the Malmquist Productivity Index (MPI) is used to measure changes in efficiency over time. For example, Li et al. [82] investigated the fuel economy of Chinese light vehicle manufacturers and Voltes-Dorta et al. [83] measured temporal changes in emissions efficiency in the Spanish automobile market and used the resulting trends to predict future emissions.

Using SFA, Oh and Shin [84] measured efficiency at the production line level, and compared different inefficiency distributions. Xu and Chen [85] investigated the innovation efficiency of Chinese new energy vehicle firms, consequently highlighting how efficiency varies across different input variables and identifying the factors that most significantly affect overall efficiency. Sur and Nandy [19] compared the efficiency of foreign direct investment-supported firms in the Indian automotive industry with that of domestic firms and identified the key determinants of the difference in efficiency. Additionally, Oh and Hildreth [86] proposed a method to improve energy efficiency in the automotive sector by combining SFA and DEA.

Although previous studies such as Neves et al. [77] and Oh and Shin [84] have strengthened the theoretical background of the automotive efficiency literature, the efficiency of the next-generation automotive industry demands further approaches that capture dynamic innovation cycles and multi-stage value networks. For instance, Tone and Tsutsui’s [87] dynamic DEA uses multi-period data to analyze efficiency trends over time, which makes this approach especially suitable for evaluating the iterative nature of AV software updates and long-term impact of R&D investment. Brandenburg and Hahn [88] also applied DEA by integrating longitudinal efficiency measurement with MPI analysis and graph‐theoretic network mapping to capture the temporal dynamics in automotive SCs. Wang et al. [89] extended the methodology by applying an MPI-based dynamic DEA framework to decompose efficiency changes and technological progress contributions among the top 20 global automobile manufacturers; they thereby enhanced the assessment of total factor productivity dynamics over time. Stefanoni and Voltes-Dorta [18] integrated environmental, social, and governance-adjusted outputs into an input-oriented DEA framework by including time trends to evaluate the efficiency of 33 global car manufacturers under environmental and sustainability pressures.

Although many studies have examined the efficiency and inefficiency of the automotive industry, research on the efficiency of next-generation vehicles is fragmented. Moreover, the majority of prior studies have used only a limited set of methodologies. To address these gaps, the current study applies SFA and MFA simultaneously to analyze and compare the efficiency of EVs and AVs in the next-generation automotive sector.

## 3. Methodology

### 3.1. Stochastic frontier analysis

SFA represents the relationship between inputs and outputs through a production function, and technical efficiency (TE) is estimated using a frontier production function that reflects the maximum achievable output for a given set of inputs. A company’s TE indicates how close its observed production level is to the frontier. Therefore, the larger the gap between a company’s production and the frontier, the lower its efficiency.

To capture changes in efficiency over time, we use Battese and Coelli’s [90] SFA model. Specifically, we estimate efficiency using Equation (1):


$$Y_{it}=f\left(x_{it},\;\beta \right)e^{V_{it}-U_{it}},\text{ i}=\text{1},\text{2},\ldots ,\text{N},\text{ t}=\text{1},\;\text{2},\ldots ,\text{ T},$$ (1)

where $Y_{it}$ denotes the output of firm *i* in period *t*, $x_{it}$ denotes the input vector of firm *i* in period *t*, and *f* is the production function with the parameter vector β. The term $V_{it}$ follows an i.i.d. normal distribution $N(\text{0},\;\sigma_{v}^{\text{2}})$ and is independent of $U_{it}$, which represents the firm’s non-negative degree of inefficiency at time *t* and is assumed to follow a half-normal distribution. From Equation (1), the TE of firm *i* at time *t* can be written as


$${TE}_{it}=e^{-U_{it}}=\frac{Y_{it}}{f(X_{it},\beta )e^{V_{it}}},\;i=\text{1},\text{2},\ldots ,N,\;t=\text{1},\text{2},\ldots ,\;T.$$ (2)

In SFA applications, the Cobb–Douglas and translog production functions are commonly used. The former is sometimes oversimplified, thereby allowing the output to be simply treated as a linear combination of inputs. Thus, we adopt the translog production function, which provides greater flexibility. Specifically, we assume a random-effect time-varying production model. Accordingly, substituting the translog form into Equation (1) gives


$$lnY_{it}=\beta_{\text{0}}+\sum_{m=\text{1}}^{\text{3}}\beta_{m}lnx_{mit}+\sum_{m=\text{1}}^{\text{3}}\sum_{k\geq m}^{\text{3}}\beta_{mk}lnx_{mit}lnx_{kit}+V_{it}-U_{it},$$ (3)

where *m* indicates the input factors, $Y_{it}$ represents the total output of firm *i* at time *t*, and $x_{i}$ is the vector of inputs, namely, capital (K), the number of employees (L), and the cost of goods sold (M). While innovation studies mainly measure the performance of technology-driven sectors using the number of patents and R&D investment (e.g., [91,92]), those on efficiency analysis using production functions primarily adopt financial data on inputs such as capital, labor, and materials and on outputs such as sales [22,27].

### 3.2. Meta-frontier analysis

In traditional SFA, a difficulty arises in comparing the TE of one company with that of other companies operating under different technologies. To overcome this limitation and enable the comparison of efficiency between groups operating under different technological conditions, we adopt MFA [20]. Based on Battese et al. [21], the meta-frontier model can be expressed as follows:


$$Y_{it}^{*}=f\left(x_{it},\;\beta^{*}\right)=e^{x_{it}\beta^{*}},\text{ i}=\text{1},\text{2},\ldots ,\text{N},\text{ N}=\sum_{i=\text{1}}^{R}N_{j},\text{ t}=\text{1},\;\text{2},\ldots ,\text{ T},\text{ s}.\text{t}.\;x_{it}\beta^{*}\geq x_{it}\beta_{\left(j\right)}\;for\;all\;j=\text{1},\text{2},\ldots ,T,$$ (4)

where *j* represents each group and $\beta^{*}$ is an unknown parameter vector subject to the constraint. From Equation (4), the meta-frontier production function lies above the production frontier of every group in all time periods, thereby enveloping each group’s frontier. For simplicity, if *f* in Equation (1) is assumed to be the form $e^{x_{it}\beta_{\left(j\right)}}$, then we can rearrange Equation (1) as follows:


$$Y_{it}=e^{-U_{it(j)}}\times \frac{e^{x_{it}\beta_{\left(j\right)}}}{e^{x_{it}\beta^{*}}}\times e^{x_{it}\beta^{*}+V_{it(j)}}.$$ (5)

Dividing both sides of Equation (5) by $e^{x_{it}\beta^{*}+V_{it(j)}}$,


$$\frac{Y_{it}}{e^{x_{it}\beta^{*}+V_{it(j)}}}=e^{-U_{it(j)}}\times \frac{e^{x_{it}\beta_{\left(j\right)}}}{e^{x_{it}\beta^{*}}}.$$ (6)

On the right hand side of Equation (6), the first term $e^{-U_{it(j)}}$ corresponds to the TE of group *j*. The second term, which is the ratio of group *j*’s frontier to the meta-frontier, is referred to as the technical gap ratio (TGR) or meta-technology ratio. Denoted as TE*, the TE of a firm relative to the meta-frontier equals the product of TE and the TGR:


$${TE}_{it}^{*}=\frac{Y_{it}}{e^{x_{it}\beta^{*}+V_{it(j)}}}={TE}_{it}\times {TGR}_{it}.$$ (7)

To estimate the parameter vector $\beta^{*}$ of the meta-frontier production function, two methods can be used: linear and quadratic programming (LP and QP, respectively). Furthermore, LP and QP aim to minimize the sum of absolute and squared deviations, respectively. The robustness of the MFA estimation results can then be confirmed using these two optimization approaches. Following Battese et al. [21], we can formally express this as follows:


$$\text{LP}:\;\underset{\beta^{*}}{\text{min}}{\text{L}}^{*}=\sum_{\text{t}=\text{1}}^{\text{T}}\sum_{\text{i}=\text{1}}^{\text{N}}\left|{\text{x}}_{\text{it}}\beta^{*}-{\text{x}}_{\text{it}}{\hat{\beta }}_{\left(\text{j}\right)}\right|,\;{\text{x}}_{\text{it}}\beta^{*}\geq {\text{x}}_{\text{it}}{\hat{\beta }}_{\left(\text{j}\right)},$$ (8)

$$\text{QP}:\;\underset{\beta^{*}}{\text{min}}{\text{L}}^{*}=\sum_{\text{t}=\text{1}}^{\text{T}}\sum_{\text{i}=\text{1}}^{\text{N}}{\left({\text{x}}_{\text{it}}\beta^{*}-{\text{x}}_{\text{it}}{\hat{\beta }}_{\left(\text{j}\right)}\right)}^{\text{2}},\;{\text{x}}_{\text{it}}\beta^{*}\geq {\text{x}}_{\text{it}}{\hat{\beta }}_{\left(\text{j}\right)}.$$ (9)

This meta-frontier framework allows us to evaluate and compare the efficiency of different groups characterized by different technical conditions.

### 3.3. Research subjects and data collection

The traditional automotive industry was once characterized by a tightly vertical ecosystem, but has witnessed the convergence across various industries and shift to EVs, which has facilitated the emergence of a more horizontal ecosystem. To define the scope of the next-generation automotive industry, we first extract firms from the database of Korea Enterprise Data (now KoDATA) to identify firms that include next-generation automotive-related keywords in their descriptions and patent portfolios. Focusing on the relevant Standard Industrial Classification codes of these firms, each firm’s name, primary products, business purpose, and registered patents are further cross-checked to determine whether they included keywords related to the next-generation automotive industry. This approach identifies approximately 16,000 firms met these criteria.

In the next step, these firms are classified into detailed subgroups. The collected observations undergo a process to structure unstructured data, focusing on patent titles and summaries. The unstructured text data are tokenized into meaningful minimum units, thereby generating tokens that represent the smallest meaningful parts of Korean words. After a preprocessing phase, including the removal of stopwords, whitespaces, and other irrelevant elements, we vectorize the data using the Term Frequency-Inverse Document Frequency (TF-IDF) technique. TF-IDF is a statistical measure that indicates the importance of a particular word in a document relative to a corpus consisting of multiple documents. This is calculated by multiplying the TF by the IDF, which represents how often a word appears in a document and how rare the word is across the document set, respectively.

Based on the TF-IDF vectorized values, this study applies K-means clustering, which is a type of unsupervised learning that groups these firms based on similar characteristics [93]. Furthermore, several alternative methodologies are considered and partially tested to cluster the structured data. Models such as latent Dirichlet allocation and Bidirectional Encoder Representations from Transformers are explored to emphasize semantic similarity. However, these approaches resulted in more fine-grained and sensitive groupings. In addition, methods such as the K-means Multi-Verse Optimizer, in which K-means clustering is combined with the multi-verse optimizer to enhance the initialization of centroids and overall clustering performance, are also reviewed [94,95]. Nonetheless, given our relatively simple dataset, K-means clustering is considered sufficient to achieve meaningful clustering results.

K-means clustering is a non-hierarchical technique that partitions data into K groups, each of which has similar characteristics among its objects. Each cluster has a centroid, and each observation is assigned to the closest of these. All observations that share the same centroid form a cluster [96]. The initial clusters are then formed by randomly selecting the K initial centroid, assigning observations to the closest centroid, and then recalculating the cluster centroid. This iterative process continues until the centroids stop moving [97].

The algorithm typically uses Euclidean distance to measure the difference between observations *i* and *j*, where *p* denotes the number of variables:


$$\begin{array}{c} d_{ij}=\sqrt{\sum {\mathrm{\backslash nolimits}}_{k=\text{1}}^{p}{\left(x_{ik}-x_{jk}\right)}^{\text{2}}}. \end{array}$$ (10)

**Fig 2:**
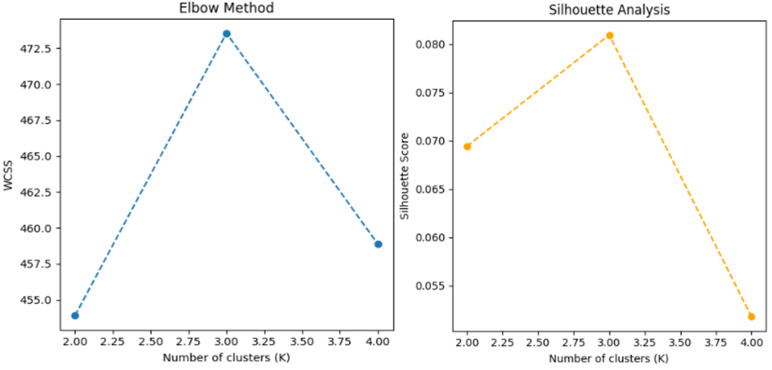
Optimal value of K: Elbow method and silhouette analysis. *Note*: K refers to the number of groups. WCSS: within-cluster sum of squares. Both approaches confirm that the optimal value of the group number equals three.

Since K-means clustering requires a priori specifying the number of clusters (K), we adopt two common approaches to determine the optimal value of K: the elbow and silhouette methods. The former computes the within-cluster sum of squares (WCSS), the squared distance between an observation and its cluster centroid, and identifies the point at which the marginal decrease in the WCSS becomes significantly smaller [98]. By contrast, the latter compares the average within-cluster distance of each data point with the average distance to the points in the nearest neighboring cluster and selects the number of clusters that yields the highest overall silhouette score [99]. Based on this analysis, we set the optimal number of clusters to three, as illustrated in Fig 2. To evaluate clustering quality, we conducted three repeated experiments with different random seeds. The average silhouette coefficient was 0.1064(±0.0087), which indicates that while cluster separation is not strong, a certain degree of thematic distinction exists within the data. The Adjusted Rand Index (ARI) was 0.4024(±0.1042) and the Normalized Mutual Information (NMI) was 0.4455(±0.0886), which suggests that the clustering structure remained relatively consistent despite variations in initialization. Overall, the dataset tends to be grouped into three primary topics; however, some documents do not exhibit clear cluster boundaries. This observation aligns with the common characteristics of unstructured text data, in which topic overlaps frequently occur. The final cluster classification corresponds to three thematic groups that define the next-generation automotive industry: the EV, AV, and general automotive technology (GAT).

**Fig 3:**
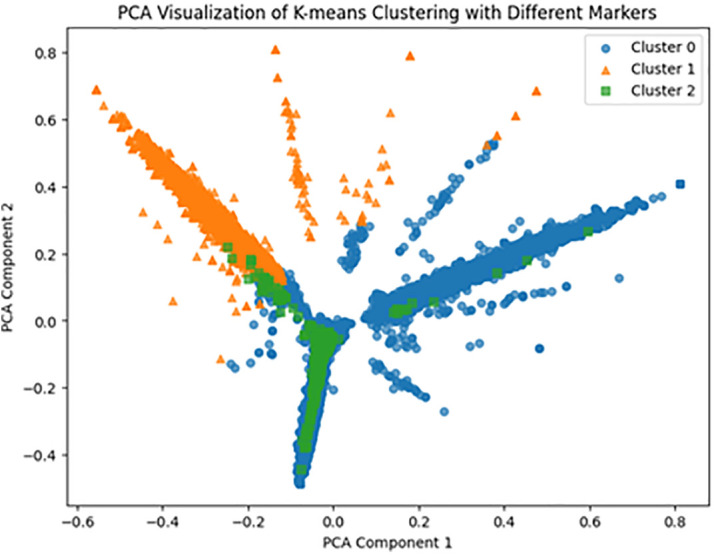
The result of PCA. *Note*: Cluster 0, 1, and 2 refer to general automotive technology, autonomous vehicle, and electric vehicle groups, respectively. PCA: principal component analysis. The result indicates that the number of groups, three, is visually confirmed.

In addition, we conduct principal component analysis (PCA), which is a dimensional reduction technique that transforms high-dimensional data into lower-dimensional data. This technique allows us to preserve the key features of the data while eliminating unnecessary information, thereby reducing dimensionality, increasing computational efficiency, and converting the data into a form suitable for visualization. This process enhances the performance of K-means clustering and helps visualize the results by transforming the data into two or three principal components. As illustrated in Fig 3, we use PCA to reduce the data to two dimensions, effectively representing three clusters, which makes this a useful tool for visually interpreting clustering results.

The optimal clustering resulted in three groups, with the top ten meaningful keywords along with their weights, average TF-IDF weights, for each summarized in Table 1. The centroids of each cluster represent the keywords characteristic of that cluster. The former are calculated as the average TF-IDF vectors of the cluster, and higher values indicate that a keyword holds relatively greater significance within it. Cluster 0 was interpreted as encompassing technologies that broadly impact the next-generation automotive industry and was labeled as the GAT group; Cluster 1, characterized by keywords such as control, prediction, LIDAR, radar, and unmanned, as the AV group; and Cluster 2, including keywords such as battery, charging, fuel cell, inverter, and electric, as the EV group.

**Table 1:** Top ten keywords and average TF-IDF weight by cluster.

Cluster	Top Keywords (TF-IDF weight)
0	Motor (0.0371), engine (0.0269), manufacture (0.0247), system (0.0243), sales business (0.0213), structure (0.0144), control method (0.0137), artificial interface (0.0131), unmanned (0.0121), device (0.012)
1	Control (0.0544), prediction (0.0525), interface (0.0429), LIDAR (0.0351), simulation (0.0299), decision (0.0204), engine (0.0202), unmanned (0.0177), motor (0.0161), radar (0.0121)
2	Battery (0.4905), charging (0.0384), system (0.0364), module (0.031), management (0.0256), fuel cell (0.0248), manufacture (0.0241), electric (0.0237), inverter (0.0179), control (0.0157)

*Note*: Cluster 0, 1, and 2 refer to general automotive technology group, autonomous vehicle group, and electric vehicle group, respectively. TF-IDF: Term Frequency-Inverse Document Frequency.

## 4. Results

### 4.1. Technical efficiency comparisons

The descriptive statistics of the sample are presented in Table 2. This study splits the data into two periods, 2017–2019 (Period 1) and 2020–2022 (Period 2), and distinguishes between the EV (ELE), AV (AUT), and GAT groups. Overall, as evidenced by the increase in the number of firms in each group and higher mean values of all the key indicators, the data indicate that this industry ecosystem has grown.

**Table 2:** Descriptive statistics of the input and output variables.

Variable	AV Group [AUT] (2017–2019)		AV Group [AUT] (2020–2022)	
	*Mean*	*Standard deviation*	*Mean*	*Standard deviation*
*Sales(Y)*	*541,366,828*	*4,037,394,532*	*541,726,128*	*4,226,868,368*
*Capital(K)*	*899,029,477*	*6,828,956,762*	*1,119,299,043*	*9,282,713,614*
*Number of employees(L)*	*503*	*3,227*	*508*	*3,471*
*Cost of goods sold(M)*	*278,555,697*	*1,973,460,551*	*344,273,846*	*2,618,101,958*
*Observations*	*191*		*227*	
**Variable**	**EV Group [ELE]** **(2017–2019)**		**EV Group [ELE]** **(2020–2022)**	
	***Mean***	***Standard deviation***	***Mean***	***Standard deviation***
*Sales(Y)*	*246,792,011*	*1,094,306,642*	*380,607,545*	*1,858,161,953*
*Capital(K)*	*398,473,975*	*2,120,591,022*	*451,041,565*	*2,375,073,210*
*Number of employees(L)*	*367*	*1,514*	*376*	*1,620*
*Cost of goods sold(M)*	*205,126,677*	*883,563,025*	*319,592,469*	*1,527,564,619*
*Observations*	*138*		*153*	
**Variable**	**General Automotive Technology Group [GAT]** **(2017–2019)**		**General Automotive Technology Group [GAT]** **(2020–2022)**	
	***Mean***	***Standard deviation***	***Mean***	***Standard deviation***
*Sales(Y)*	*505,686,214*	*3,037,759,594*	*528,125,683*	*3,521,970,105*
*Capital(K)*	*627,836,320*	*4,553,287,478*	*649,134,205*	*4,938,167,703*
*Number of employees(L)*	*616*	*3,875*	*449*	*2,347*
*Cost of goods sold(M)*	*440,901,766*	*2,591,406,328*	*457,661,064*	*2,978,762,715*
*Observations*	*1,310*		*1,538*	

*Note*: AV: autonomous vehicle; EV: electric vehicle.

The coefficients of the translog production function estimated using SFA and MFA for Periods 1 and 2, are reported in Tables 3 and 4, respectively. From the SFA estimation results for all periods, the following interpretations can be drawn. The coefficients of the AV group are not consistently significant, which indicates a sector in a period of initial growth, while those of the number of employees and cost of goods sold of the EV group are significant and positive, thereby suggesting a slightly more robust ecosystem than the AV group. In the GAT group, while the coefficients of capital and the number of employees are significant, the cost of goods sold displays a negative and positive coefficient for the linear and squared terms, respectively, ultimately exhibiting a U-shaped pattern. This indicates that, akin to traditional manufacturing, technologies commonly adopted in the automotive industry have been used for a sufficiently long time. Furthermore, the MFA results reveal the coefficients for both the LP and the QP approaches. The findings from these two approaches show consistent patterns regarding direction and value, thereby demonstrating a robust form of meta-frontier production function for the following analysis.

**Table 3:** Estimation results of SFA and MFA in Period 1 (2017–2019).

Variable	SFA			MFA	
	*AUT*	*ELE*	*GAT*	*LP*	*QP*
Constant	−0.64 (0.979)	0.192^***^ (0.392)	8.756^***^ (1.083)	5.103	6.544
$ln\;x_{k}$	−0.054 (0.501)	0.249^***^ (0.092)	0.916^***^ (0.198)	0.572	0.738
$ln\;x_{l}$	−0.69^***^ (0.236)	0.101^**^ (0.705)	1.5^***^ (0.223)	0.231	0.441
$ln\;x_{m}$	1.381^***^ (0.499)	0.833^***^ (0.549)	−1.223^***^ (0.134)	−0.177	−0.569
${\left(ln\;x_{k}\right)}^{\text{2}}$	0.028 (0.041)	−0.042^*^ (0.056)	−0.015 (0.012)	0.043	0.012
${\left(ln\;x_{l}\right)}^{\text{2}}$	−0.036^**^ (0.017)	−0.006^*^ (0.040)	0.087^***^ (0.014)	0.001	0.01
${\left(ln\;x_{m}\right)}^{\text{2}}$	−0.003 (0.008)	−0.024^**^ (0.023)	0.087^***^ (0.005)	0.101	0.097
$ln\;x_{k}\times ln\;x_{l}$	0.006 (0.047)	0.045^*^ (0.099)	0.019 (0.019)	0.06	0.093
$ln\;x_{l}\times ln\;x_{m}$	−0.042 (0.047)	0.062^*^ (0.058)	−0.02 (0.032)	−0.124	−0.079
$ln\;x_{m}\times ln\;x_{k}$	0.053 (0.037)	−0.037^*^ (0.065)	−0.148^***^ (0.014)	−0.075	−0.127

*Note*: ***, **, and * = *p* < 0.01, *p* < 0.05, *p* < 0.1, respectively. Standard errors are in parentheses. ${\text{x}}_{\text{k}}$, ${\text{x}}_{\text{l}}$, and ${\text{x}}_{\text{m}}$ are the inputs: capital, the number of employees, and the cost of goods sold, respectively. SFA: stochastic frontier analysis; MFA: meta-frontier analysis. LP and QP refer to the values estimated by linear and quadratic programming, respectively. Group indicators are defined in the note for Table 2.

**Table 4:** Estimation results of SFA and MFA in Period 2 (2020–2022).

Variable	SFA			MFA	
	*AUT*	*ELE*	*GAT*	*LP*	*QP*
Constant	−4.949^***^ (1.808)	9.856^***^ (1.866)	10.618^***^ (0.943)	12.826	12.135
$ln\;x_{k}$	1.489^***^ (0.523)	−1.106^***^ (0.372)	0.677^***^ (0.155)	0.098	0.214
$ln\;x_{l}$	−1.839 (0.378)	0.933^**^ (0.383)	1.257^***^ (0.145)	1.319	1.257
$ln\;x_{m}$	0.605 (0.451)	0.848^***^ (0.248)	−1.096^***^ (0.086)	−0.796	−0.789
${\left(ln\;x_{k}\right)}^{\text{2}}$	−0.029 (0.032)	0.066^***^ (0.022)	−0.032^***^ (0.008)	0.031	0.014
${\left(ln\;x_{l}\right)}^{\text{2}}$	−0.117^***^ (0.027)	0.02 (0.024)	0.034^***^ (0.009)	0.038	0.034
${\left(ln\;x_{m}\right)}^{\text{2}}$	0.049^***^ (0.019)	0.02^**^ (0.009)	0.052^***^ (0.003)	0.095	0.083
$ln\;x_{k}\times ln\;x_{l}$	0.214^***^ (0.053)	−0.088^**^ (0.033)	0.025^*^ (0.015)	0.015	0.025
$ln\;x_{l}\times ln\;x_{m}$	−0.073^**^ (0.036)	−0.045^*^ (0.025)	0.028^***^ (0.009)	−0.069	−0.045
$ln\;x_{m}\times ln\;x_{k}$	−0.05 (0.047)	0.029^*^ (0.023)	−0.108^***^ (0.01)	−0.109	−0.109

*Note*: ***, **, and * = *p* < 0.01, *p* < 0.05, *p* < 0.1, respectively. Standard errors are in parentheses. ${\text{x}}_{\text{k}}$, ${\text{x}}_{\text{l}}$, and ${\text{x}}_{\text{m}}$ are the inputs: capital, the number of employees, and the cost of goods sold, respectively. SFA: stochastic frontier analysis; MFA: meta-frontier analysis. LP and QP refer to the values estimated by linear and quadratic programming, respectively. Group indicators are defined in the note for Table 2.

Before conducting the MFA, to determine whether all groups share an identical production technology is important. If the production functions are the same across groups, estimation of separate frontiers for each group is not necessary. To test this, we estimated a pooled SFA model, where all observations are combined, as the restricted model, and individual group-specific frontier models were estimated as the unrestricted models for the likelihood ratio (LR) test. The value of LR is calculated by 2(logLu−logLr), where *logLr* and *logLu* denotes the log-likelihood value of the restricted model and the sum of the log-likelihood values from the unrestricted group-specific models, respectively. The value of LR is 274.881, which indicates the hypothesis that all groups share identical production function parameters can be rejected at the 1% significance level. This result confirms that the group-specific SFA model is more appropriate than the pooled model, and that each group exhibits distinct production and efficiency structures.

The TE values obtained through SFA are reported in Table 5. In Period 1, the GAT group displays the highest mean TE, followed by the EV and AV groups. However, in Period 2, the TE values of both the AV and the EV groups increase, while the TE value of the GAT group slightly decreases. One possible interpretation of these changes from Period 1 to Period 2 is that recent technological innovation in AVs and EVs has driven TE gains, thereby outpacing the TE of the GAT group.

**Table 5:** Technical efficiency across autonomous vehicles, electric vehicles, and general automotive technology groups obtained through SFA for Period 1 (2017–2019) and Period 2 (2020–2022).

Period 1	AUT	ELE	GAT
Mean	0.717	0.763	0.837
Standard deviation	0.091	0.093	0.115
Minimum	0.426	0.093	0.071
Maximum	0.956	0.971	0.989
**Period 2**	**AUT**	**ELE**	**GAT**
Mean	0.867	0.864	0.815
Standard deviation	0.093	0.11	0.122
Minimum	0.351	0.408	0.113
Maximum	0.978	0.982	0.986

*Note*: Group indicators are defined in the note for Table 2.

**Fig 4:**
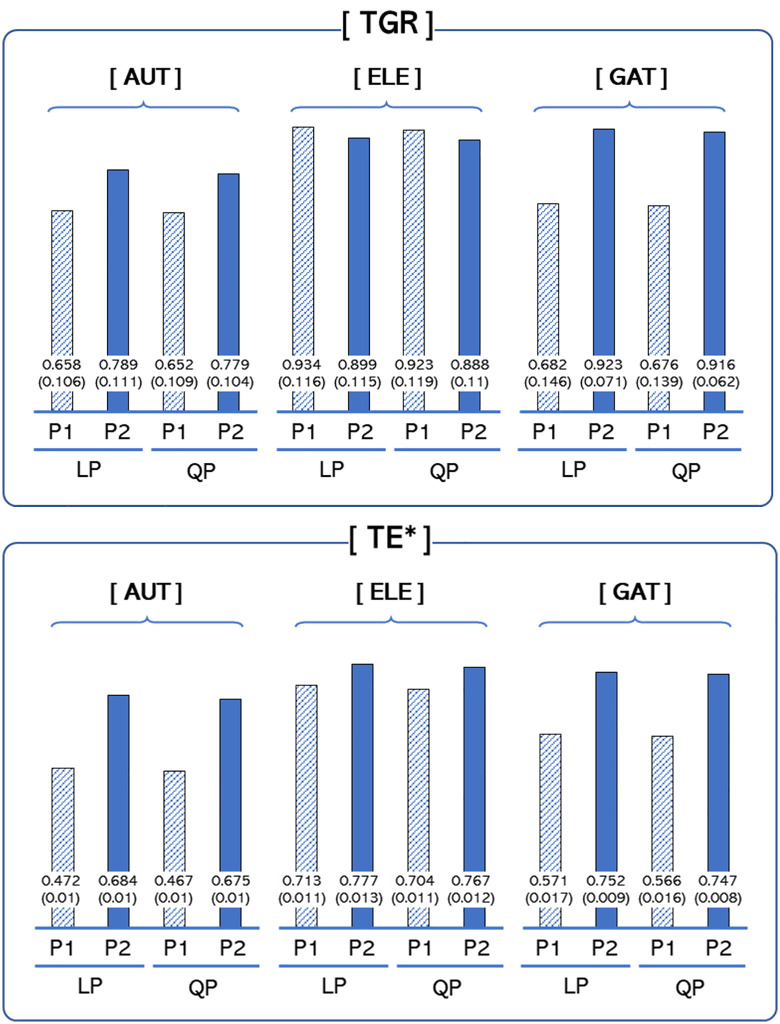
Efficiency dynamics of meta-frontier analysis. *Note*: LP and QP refer to the values estimated by linear and quadratic programming, respectively. P1 and P2 refer to the period from 2017 to 2019 and from 2020 to 2022, respectively. Standard deviations are in parentheses. TGR: technology gap ratio. TE*: technical efficiency of a firm relative to the meta-frontier production function. Group indicators are defined in the note for Table 2. The results reveal a gradual increase in efficiency from P1 to P2.

The TGR and TE* values obtained through MFA are reported in Tables 6 and 7, respectively, and the efficiency dynamics from MFA are illustrated in Fig 4. The TGR indicates how much the stochastic frontier production function has shifted relative to the meta-frontier production function. While a high TGR indicates more active technological innovation, a high TGR but relatively low TE* value often indicates unbalanced growth in the group, with a few highly innovative firms increasing the stochastic frontier, thus leaving most firms lagging behind. In Period 1, the EV group exhibits the highest mean TE* value, followed by the GAT and AV groups. This result is consistent with the TGR results, which means that EVs experienced active technological innovation during that period. In Period 2, the EV group again records the highest TE* value, followed by the GAT and AV groups. However, when comparing the mean TGRs in Period 2, the GAT group experiences the greatest improvement in technological innovation. A possible explanation is that this group made considerable investments in technological innovation, which suppressed its overall efficiency in the short term despite its progress in shifting the production frontier.

**Table 6:** Technology gap ratio across autonomous vehicles, electric vehicles, and general automotive technology groups obtained through MFA for Period 1 (2017–2019) and Period 2 (2020–2022).

	Period 1	AUT	ELE	GAT
LP	Mean	0.658	0.934	0.682
	Standard deviation	0.106	0.116	0.146
	Minimum	0.233	0.256	0.003
	Maximum	1	1	0.884
QP	Mean	0.652	0.923	0.676
	Standard deviation	0.109	0.119	0.139
	Minimum	0.232	0.3	0.003
	Maximum	1	1	0.931
	**Period 2**	**AUT**	**ELE**	**GAT**
LP	Mean	0.789	0.899	0.923
	Standard deviation	0.111	0.115	0.071
	Minimum	0.292	0.196	0.128
	Maximum	1	1	1
QP	Mean	0.779	0.888	0.916
	Standard deviation	0.104	0.11	0.062
	Minimum	0.313	0.233	0.271
	Maximum	1	1	1

*Note*: LP and QP refer to the values estimated by linear and quadratic programming, respectively. Group indicators are defined in the note for Table 2.

**Table 7:** Technical efficiency relative to the meta-frontier across autonomous vehicles, electric vehicles, and general automotive technology groups obtained through MFA for Period 1 (2017–2019) and Period 2 (2020–2022).

	Period 1	AUT	ELE	GAT
LP	Mean	0.472	0.713	0.571
	Standard deviation	0.01	0.011	0.017
	Minimum	0.099	0.024	0
	Maximum	0.956	0.971	0.874
QP	Mean	0.467	0.704	0.566
	Standard deviation	0.01	0.011	0.016
	Minimum	0.099	0.028	0
	Maximum	0.956	0.971	0.921
	**Period 2**	**AUT**	**ELE**	**GAT**
LP	Mean	0.684	0.777	0.752
	Standard deviation	0.01	0.013	0.009
	Minimum	0.102	0.08	0.014
	Maximum	0.978	0.982	0.986
QP	Mean	0.675	0.767	0.747
	Standard deviation	0.01	0.012	0.008
	Minimum	0.1	0.095	0.031
	Maximum	0.978	0.982	0.986

*Note*: LP and QP refer to the values estimated by linear and quadratic programming, respectively. Group indicators are defined in the note for Table 2.

As shown in Table 7, the TE* values of all three groups increase from Period 1 to Period 2, with the AV group displaying the largest increase. This increase in the TE* value for this group exceeds that in the TGR, which suggests that specific innovations in the AV sector may have accelerated efficiency gains by strengthening the group’s overall technological base. Furthermore, the GAT group also exhibits substantial improvements in efficiency, which implies that the industry’s broad expansion is being driven by growth in several next-generation automotive sectors. Finally, all these findings are consistent with both LP and QP. The efficiency rankings are identical, and the values for each measure are also comparable, thereby providing reliable results. This indicates the robustness of MFA.

### 4.2. Robustness check of the efficiency differences across groups

To check the robustness of the efficiency differences and examine the significance of the factors influencing efficiency, we adopt two approaches. First, we use a two-way analysis of variance (ANOVA) to examine the effects of period and group on *TGR_LP*. The two-way ANOVA results reveal the significant main effects of period (F(1, 3551) = 582.15, p < .001) and group (F(2, 3551) = 140.14, p < .001) as well as the highly significant interaction between them (F(2, 3551) = 899.69, p < .001). The model accounts for approximately 67.2% of the total variance in *TGR_LP* (R² = .672) and this significant interaction suggests that the effect of group membership on *TGR_LP* varies by period.

Second, we conduct a Tobit analysis after controlling other relevant variables. Given that the derived TGR is constrained within a bounded range between zero and one, a censored regression model such as a Tobit model is suitable for our analysis. Factors expected to influence the technological gap by group include period, capital, the number of employees, and the cost of goods sold. In addition, a time trend variable reflecting the degree of technological progress is incorporated [27,100]. To verify the robustness of the results, we compare two dependent variables, *TGR_LP* and *TGR_QP*, using Equation (11):


$${𝐓𝐆𝐑}_{i}=\text{CONSTANT}+\beta_{\text{1}}{𝐗}_{it}+{\beta_{\text{2}}\text{timetrend}}_{it}+\beta_{\text{3}}DUM\text{1}+\beta_{\text{4}}DUM\text{2}+\epsilon_{i}.$$ (11)

As presented in Table 8, the group dummy variables reach significance, thereby confirming that group differences affect the technology gap. However, the EV and GAT groups displayed negative coefficients compared to the benchmarking AV group in Period 1. This suggests that, when controlling for input factors, firms in the AV group were closer to the meta-frontier production function while those in other groups are more dependent on input factors. For example, in the early stages of transition, a large labor force could have positively influenced efficiency. Conversely, the analysis results for Period 2 align with the MFA results, which indicates that the technological level of both groups exceeded that of the AV group. While the positive effect of labor has disappeared, capital, as measured by QP, has emerged as a positive factor, ultimately emphasizing the importance of investment in innovation. Following the establishment of a technological development trajectory, the efficiency gap between groups became clearly identifiable, consequently exhibiting the dynamics of technological innovation while also demonstrating that the transition to the next-generation automotive industry is stable and robust. By contrast, the time trend variable does not reach significance, which suggests that the effect of technological advancement over time has already been offset because the analysis was divided into Periods 1 and 2. Overall, the existence of group-level effects is confirmed.

**Table 8:** Tobit regression results for Period 1 (2017–2019) and Period 2 (2020–2022).

Variable	Period 1		Period 2	
	*LP*	*QP*	*LP*	*QP*
Time trend	0.000(0.002)	0.000(0.002)	0.000(0.002)	0.000(0.002)
Log(capital)	−0.059(0.004)	−0.051***(0.003)	−0.002(0.003)	0.02***(0.002)
Log(employee)	0.034***(0.004)	0.021***(0.003)	−0.010***(0.003)	−0.017***(0.002)
Log(cost)	−0.005***(0.002)	−0.001(0.002)	0.007***(0.002)	−0.007***(0.002)
Dum1	−0.234***(0.009)	−0.231***(0.009)	0.109***(0.008)	0.109***(0.008)
Dum2	−0.289***(0.006)	−0.285***(0.006)	0.134***(0.006)	0.138***(0.053)
Constant	1.857***(0.036)	1.705***(0.035)	0.749***(0.027)	0.617***(0.024)
Log likelihood	1747.574	1776.863	2103.889	2329.313

Note: ***, **, and * = p < 0.01, p < 0.05, p < 0.1, respectively. Standard errors are in parentheses. LP and QP refer to the values estimated by linear and quadratic programming, respectively.

## 5. Concluding remarks

Although the automotive SC has traditionally been dominated by a vertically integrated structure focused on ICE vehicles, the SC is being reorganized into a horizontal ecosystem as ICT, software, and related high-tech firms enter the market. The number of those firms has increased rapidly since around 2010, when next-generation automobiles began to receive attention, ultimately driving the rise in vehicles such as EVs and AVs. While the existing automotive SC centered on OEMs and connected hierarchical suppliers, the next-generation automotive industry maintains a more open ecosystem in which multiple seller–buyer relationships coexist. In this new environment, firms collaborate with various partners and easily cross heterogeneous boundaries.

Therefore, this study empirically analyzed the transition of the automotive industry from a vertical SC to a horizontal ecosystem. Here, we brought the research questions that we proposed. First, how can the next-generation automotive ecosystem be objectively defined and categorized based on firm-level technological activities? To propose the next-generation automotive ecosystem, we used an unsupervised learning approach, K-means clustering based on firm-level patent data, and identified three groups: EV, AV, and GAT. Second, how different is the growth potential and development trajectory for each ecosystem over time? By using MFA, which enables comparisons among different groups, our analysis revealed that the EV ecosystem led the initial growth, while the GAT ecosystem continued to make steady progress. Later, the AV ecosystem exhibited remarkable technological innovation and efficiency gains. By addressing these results, we can briefly summarize the contributions of this study. First, this study defined the next-generation automotive industry as an evolving and dynamic sector. Second, this study categorized firms based on the key characteristics of such automotive technologies. Finally, this study compared each ecosystem to examine the improvement in efficiency through innovation, thereby revealing its growth prospects and development trajectories.

This study has the following limitations: Although technological innovation in industries undergoing rapid change is a crucial factor, external factors such as market dynamics (e.g., consumer adoption) and government policies (e.g., subsidies) also determine the degree of innovation and efficiency. For example, while the EV sector in Korea was able to grow in its early days, such market growth could not be maintained, partly due to several battery issues (e.g., explosions and accidents). In addition, one large firm (i.e., Tesla) led technological innovation in AVs and firms such as Hyundai quickly followed, consequently increasing the innovativeness of the entire industry. Given our focus on measuring efficiency using financial data, the fact that such factors are not incorporated into the production function model remains a limitation. Thus, a future research direction could be to construct a model including these factors and adopt additional panel analysis. Finally, this study heavily relies on limited financial variables such as sales, capital, and cost of goods sold. Consequently, from the perspective of technological innovation, variables potentially affecting efficiency, including market dynamics and government policy, may not have been sufficiently incorporated in the modeling framework. This may cause the driving forces of innovation not to be fully recognized when interpreting efficiency. Using different approaches such as DEA or a different form of production function that incorporates novel variables, future research should pursue in-depth analysis from an innovation perspective.

The sector will continue to evolve toward eco-friendly, socially responsible, and economically viable solutions. Another promising research direction could be to map the networks among these automotive companies to provide a more concrete perspective of the next-generation automotive industry. Tracking the evolution of this ecosystem over time and forecasting its evolution are essential to guide technological development and foster industry growth in related domains.
